# MicroRNA-30a Regulation of Epithelial-Mesenchymal Transition in Diabetic Cataracts Through Targeting SNAI1

**DOI:** 10.1038/s41598-017-01320-3

**Published:** 2017-04-25

**Authors:** Lu Zhang, Ye Wang, Wenfeng Li, Panagiotis A. Tsonis, Zhiyuan Li, Lixin Xie, Yusen Huang

**Affiliations:** 1 Department of Ophthalmology, School of Medicine, Shandong University, Jinan, China; 2 State Key Laboratory Cultivation Base, Shandong Provincial Key Laboratory of Ophthalmology, Shandong Eye Institute, Shandong Academy of Medical Sciences, Qingdao, China; 3 Qingdao University Affiliated Hospital, Qingdao, China; 4 Department of Biology and Center for Tissue Regeneration and Engineering, University of Dayton, Dayton, Ohio United States; 5 Central Laboratory of the Second Affiliated Hospital, Medical College of Qingdao University, Qingdao, China; 6 Affiliated Hospital of Qingdao University, Qingdao, China

## Abstract

Epithelial-mesenchymal transition (EMT) is a highly conserved and fundamental process in development, fibrosis, and metastasis. During the process, epithelial cells lose their morphology and transcriptional program, and transdifferentiate to mesenchymal cells. It has been reported that lens epithelial cells undergo EMT during cataract formation, and regulation of microRNAs on genes is associated with lens development. However, the molecular mechanisms of this regulation in diabetic cataract still need to be investigated. In the present study, the expression of E-cadherin was downregulated, while the expression of alpha-SMA and vimentin was upregulated in diabetic cataract tissues and the *in vitro* model, suggesting the involvement of EMT in diabetic cataract formation. Results of miRNA profiling demonstrated that miR-30a was markedly downregulated in diabetic cataract tissues. Overexpression of miR-30a-5p decreased SNAI1, a known modulator of EMT, and the expression of vimentin and alpha-SMA in our diabetic cataract model *in vitro*. It is concluded that EMT is involved in human diabetic cataract, and upregulation of miR-30a can repress EMT through its targeting of SNAI1 in lens epithelial cells, which make miR-30a a novel target of therapeutic intervention for human diabetic cataract.

## Introduction

Cataract or lens opacification is one of the major reasons causing visual impairment and blindness^1^. Diabetic cataract often occurs in earlier age of patients with high blood glucose and progresses fast^2–4^. Currently, the only treatment is to remove the opaque lens and implant a synthetic intraocular lens through surgery. It is significative to develop novel therapies for prevention and management of diabetic cataract.

Lens epithelial cells (LECs) are responsible for differentiation of lens fibers throughout the life of a lens. Correct differentiation and morphology of LECs maintain the normal transparency of the lens, while pathological changes in development, proliferation, differentiation, and apoptosis in LECs may lead to cataract^5–7^. The presence of epithelial-mesenchymal transition (EMT) during epithelial cell differentiation into fiber cells has been reported in the progress of cataract formation^8–13^. MicroRNAs (miRNAs) are a group of noncoding small RNAs and have been found to be associated with cell differentiation, proliferation, and apoptosis^14–16^. We previously detected that miR-204-5p could directly regulate EMT by targeting SMAD4 in posterior capsule opacification (PCO) known as a complication of cataract surgery^17^. In the study, an established capsular bag model cultured *in vitro*, which can faithfully recapitulate conditions *in vivo*, was employed.

Herein, we identified the ectopic expression of miR-30a in diabetic cataract lenses and further investigated the inverted regulation of miR-30a in demotion of EMT by targeting SNAI1 in LECs. Thus, we ascertained that miR-30a may play a role in the progress of diabetic cataract formation.

## Results

### Expression of miR-30a and EMT in human diabetic cataract LECs

**Figure 1:**
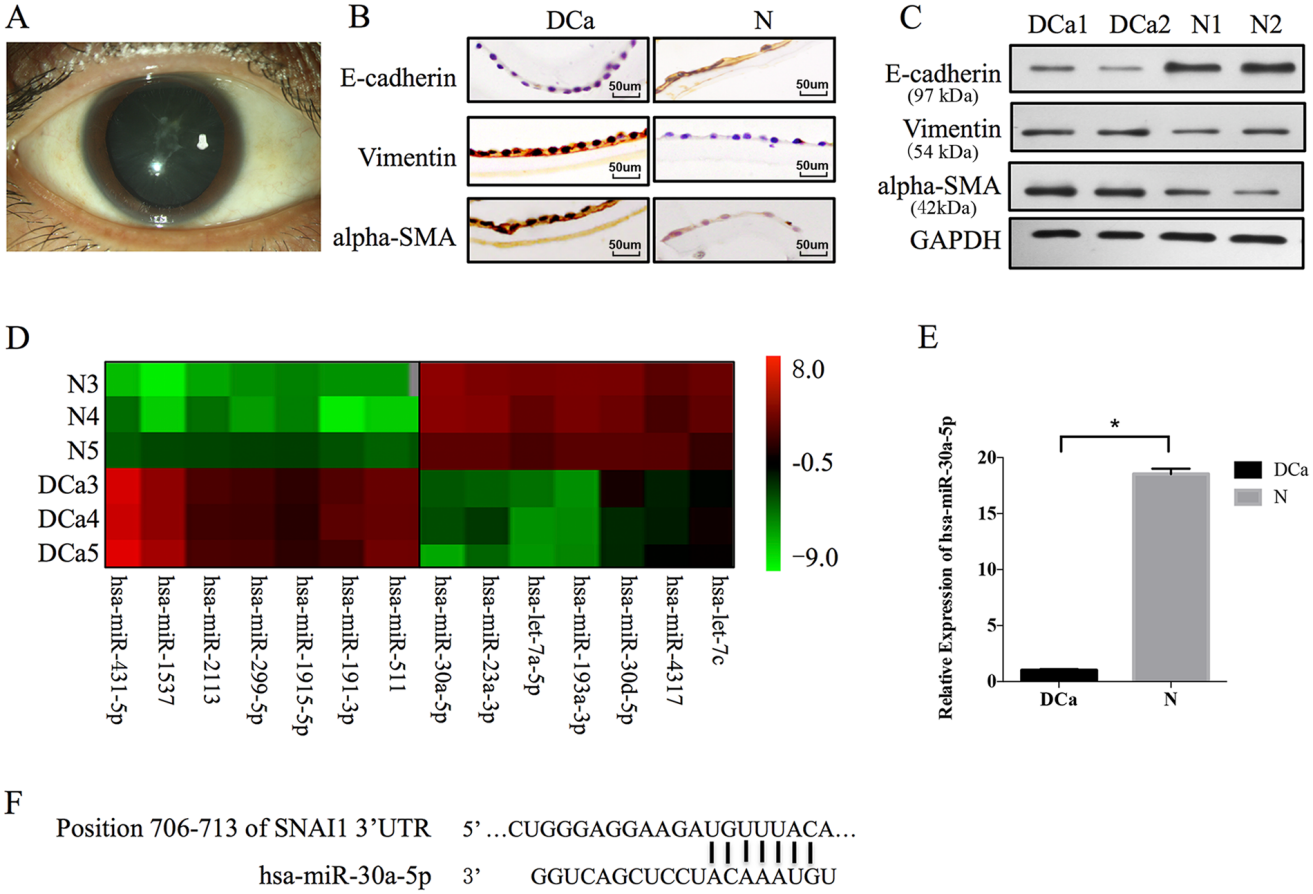
Expression of the epithelial marker (E-cadherin) and EMT markers (vimentin and alpha-SMA) and miRNA profiles in the normal and diabetic cataract lens epithelial cells (LECs). (**A**) Opacity of lens subcapsular tissues in diabetic cataract patients. (**B**) Immunohistochemistry demonstrated a high expression of E-cadherin and a low expression of vimentin and alpha-SMA in normal LECS, and a significantly downregulated expression of E-cadherin and an upregulated expression of vimentin and alpha-SMA in diabetic cataract LECs. (**C**) Western blotting for quantification of the protein expression (top, data from the gels; bottom, normalization to GAPDH). Two normal samples (N1 and N2) and two diabetic cataract samples (DCa1 and DCa2) were included. (**D**) Hierarchical clustering was performed [Rows, miRNA; Column, three normal samples (N3, N4, and N5) and three diabetic cataract samples (Dca3, Dca4, and Dca5)]. A total of 272 miRNAs were identified as having an altered expression more than 2-fold between the diabetic cataract and normal LECs, and 14 regulated miRNAs were depicted in this figure. The red color indicates the high expression, and the green color denotes the low expression. (**E**) qRT-PCR was performed by using the same extracted total RNA for the microarray analysis to validate the microarray data. The relative amount of hsa-miR-30a-5p was normalized to the U6 expression. The relative expression levels of hsa-miR-30a-5p were downregulated significantly in the diabetic cataract LECs (N as control, *P < 0.05). Significant differences are indicated by t-test (*P < 0.05). (**F**) Position of the miR-30a-5p target sequence in the 3′-UTR of SNAI1 mRNA.

In patients with diabetic cataract, opacity of lens subcapsular tissues was observed (Fig. 1A). By immunohistochemistry (Fig. 1B), the protein expression of E-cadherin was found to be higher in normal lens tissues (N) than in diabetic cataract tissues (DCa), while alpha-smooth muscle actin (alpha-SMA, a late marker of EMT) and vimentin showed a higher expression in DCa compared with N. Two normal samples (N1 and N2) and two diabetic cataract samples (DCa1 and DCa2) were used to perform Western blotting (Fig. 1C). The results revealed a decreased expression of E-cadherin in DCa1 and DCa2 and an increased expression of alpha-SMA and vimentin when compared to N1 and N2. Both immunohistochemistry and Western blot analyses suggested that EMT was a significant event in LECs of patients with diabetic cataract.

To investigate whether miRNAs were differentially expressed in normal and diabetic cataract LECs, a miRNA microarray analysis was performed using on capsular bags from three normal lenses (N3, N4, and N5) and three diabetic cataract lenses (DCa3, DCa4, and DCa5). Based on the pooled data from each group, the results showed that 73 miRNAs in the diabetic cataract LECs were unregulated more than two folds (N3, N4, and N5 as control), while 199 miRNAs were downregulated more than two folds. The expression of miR-30a-5p was downregulated 180-fold. The part of the heatmap is presented in Fig. 1D.

MiR-30a was further investigated because it was predicted to target SNAI1 associated with EMT. To confirm whether there was significant difference in the expression of hsa-miR-30a between the normal and diabetic cataract LECs, quantitative RT-PCR (qRT-PCR) was employed using the RNA of the miRNA microarray study. The hsa-miR-30a expression was downregulated significantly in the diabetic cataract LECs compared with the normal ones (P < 0.05, Fig. 1E). According to the bioinformatics database (available at http://www.targetscan.org/), miR-30a targets SNAI1 3′-UTR (706–713) (Fig. 1F).

### Diabetic cataract model *in vitro*

**Figure 2:**
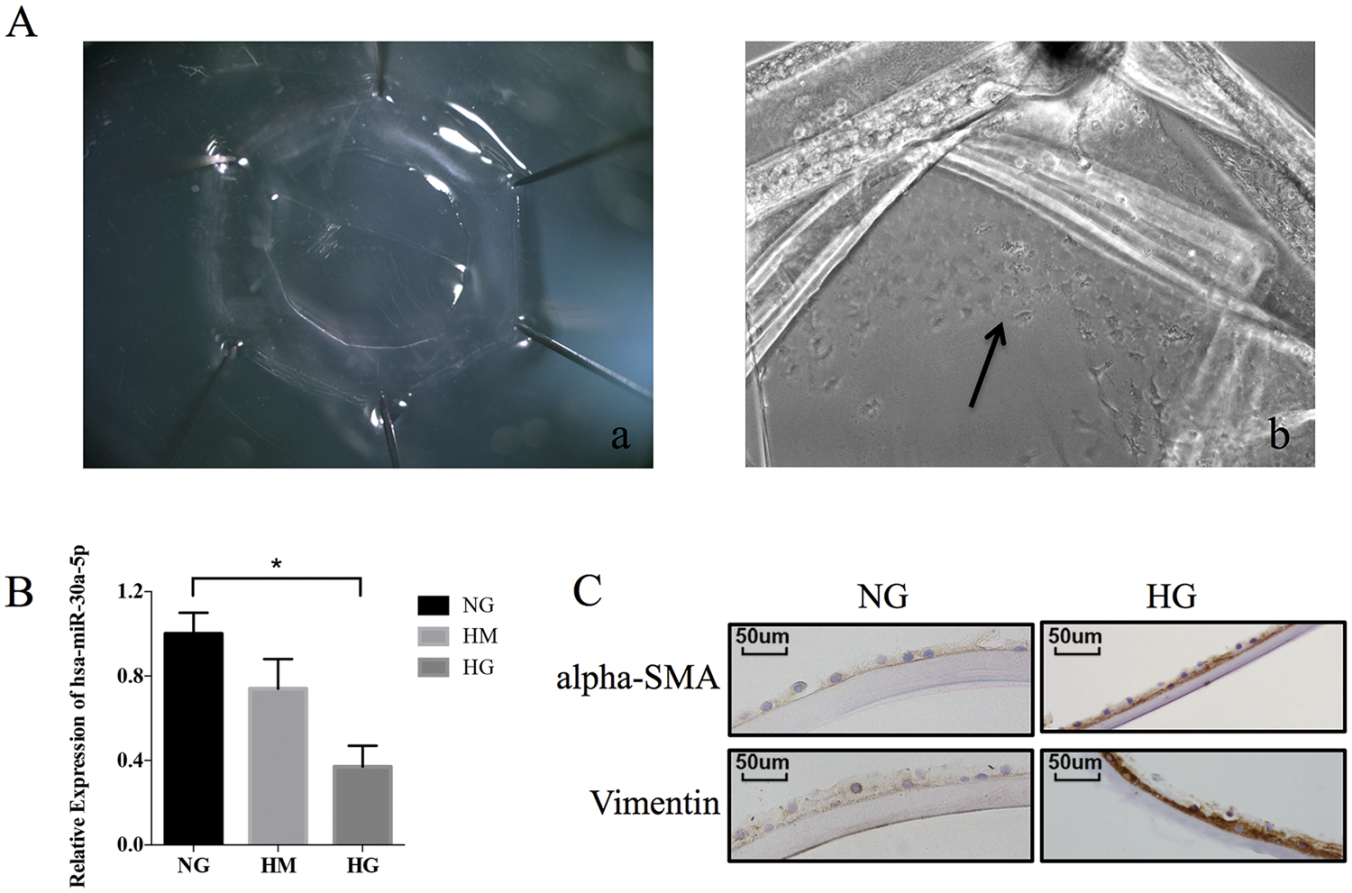
Human donor capsular bags as a diabetic cataract model *in vitro*. (**A**) Capsular bags were immersed in the culture medium: (a) immediately after pinning; (b) after 24 hours, when the LECs were observed to be migrating (arrow). (**B**) The expression of miR-30a-5p was detected by qRT-PCR. MiR-30a-5p increased in the presence of HG (*P < 0.05, NG as control, N = 1), instead of HM (osmotic control). (**C**) Immunohistochemistry analysis was performed to detect the protein expression of EMT markers. The staining intensity of vimentin and alpha-SMA expression increased in HG compared to NG.

The lens capsular bags were cultured in DMEM-F12 with 10% fetal bovine serum containing 5 mM D-glucose (normal glucose, NG), 25 mM D-glucose (high glucose, HG), or 5 mmol/L D-glucose plus 20 mmol/L mannitol (high mannitol, HM). The results of qRT-PCR in LECs showed that the expression of miR-30a-5p was increased in HG conditions (NG as control; P < 0.05, Fig. 2B). In the *in vitro* model, the expression of EMT markers was also detected by immunohistochemistry (Fig. 2C). The staining intensity of vimentin and alpha-SMA protein expression increased in HG compared to NG, suggesting that our diabetic cataract model *in vitro* adequately simulated the states *in vivo*.

### MiR-30a targeted SNAI1 in the diabetic cataract model *in vitro*

**Figure 3:**
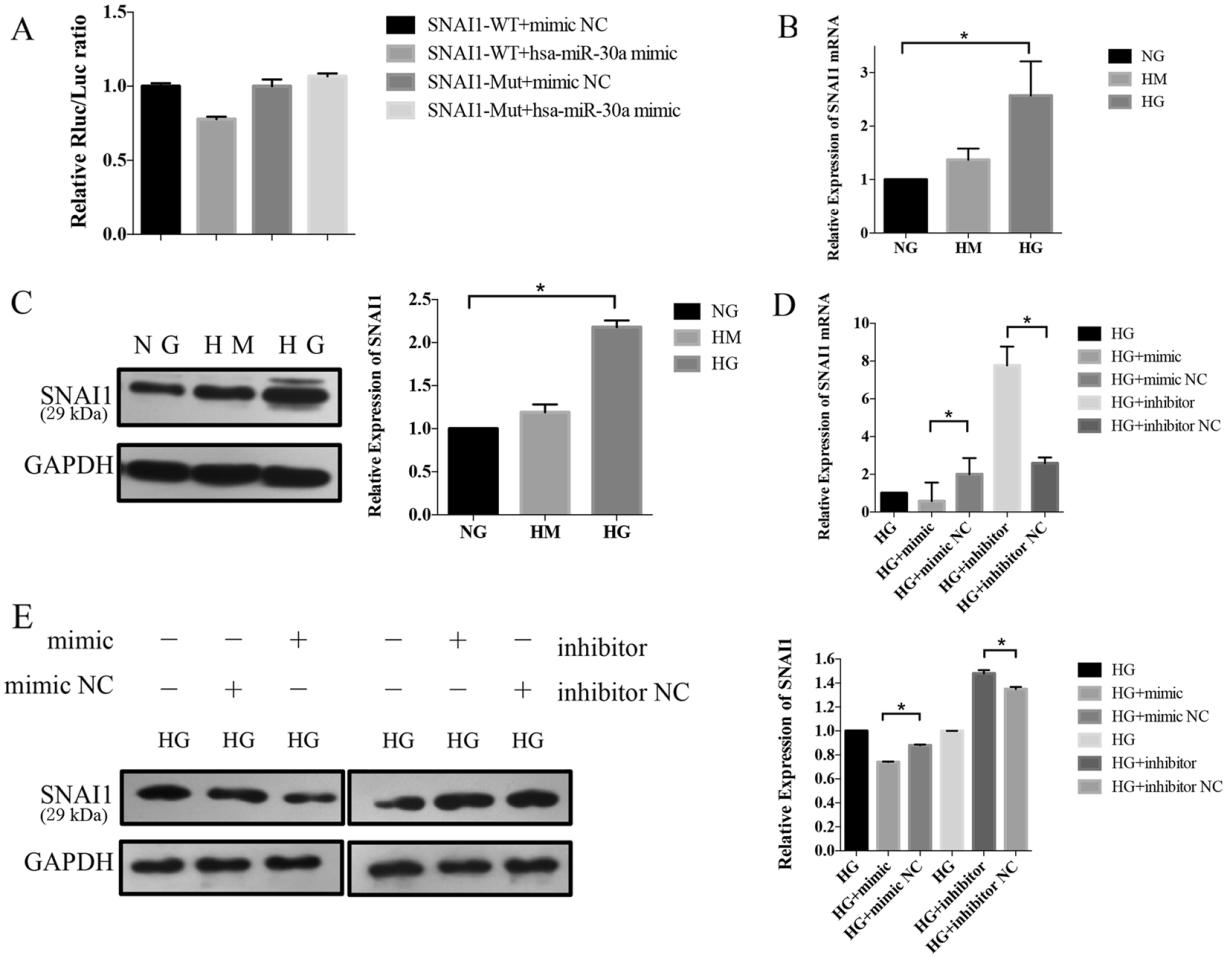
MiR-30a-5p directly targeted and regulated SNAI1 in the diabetic cataract model *in vitro*. (**A**) A dual luciferase reporter assay. When pmiR-RB-REPORT-SNAI1-3′UTR and miR-30a-5p mimic were cotransfected, a significant decrease in relative luciferase activity was observed. The mutation of the perfectly complementary sites in the SNAI1-3′UTR disrupted the interaction between miR-30a-5p and Snai11 and abolished the suppressive effect. (**B**) The mRNA expression of SNAIl. qRT-PCR showed the SNAI1 mRNA expression increased significantly in HG (NG as control, *P < 0.05, N = 1). (**C**) The protein expression of Snail in Western blot. Left: The representative gel results (top, data from the gels; bottom, normalization to GAPDH); Right: The normalized graphs results. The result showed an increased expression of SNAI1 in HG conditions (NG as control, *P < 0.05, N = 2). (**D**) Results of qRT-PCR in the transfected LECs. There were five groups in this part: 1, capsular bags cultured in HG for 3 days were used as the control group; 2, the cells were transfected with a mimic negative control (NC) of miR-30a-5p; 3, the cells were transfected with a mimic of miR-30a-5p; 4, the cells were transfected with an inhibitor of miR-30a-5p; and 5, the cells were transfected with an inhibitor NC of miR-30a-5p. SNAI1 mRNA levels were significantly increased by the miR-30a-5p mimic (*P < 0.05, mimic NC as control, N = 1) and decreased by the miR-30a-5p inhibitor (*P < 0.05, inhibitor NC as control, N = 1). (**E**) Results of Western blotting in the transfected LECs (top, data from the gels; bottom, normalization to GAPDH). SNAI1 protein expression was decreased by transfection with the miR-30a-5p mimic (*P < 0.05, mimic NC as control, N = 2) but restored by transfection with the miR-30a-5p inhibitor (*P < 0.05, inhibitor NC as control, N = 2) in the LECs significantly.

A dual luciferase reporter assay was performed to test the direct link between miR-30a and SNAI1 (Fig. 3A). When pmiR-RB-REPORT-SNAI1-3′UTR and miR-30a mimic were cotransfected, the relative luciferase activity decreased significantly. Moreover, mutation of the perfectly complementary sites in the SNAI1-3′UTR abolished the suppressive effect of miR-30a. These results confirmed that miR-30a-5p could directly target and regulate SNAI1. In the *in vitro* model, the SNAIl mRNA and protein expression increased significantly in HG (NG as control, Fig. 3B and C). After the capsular bags were cultured in HG conditions for three days, they were transfected with a miR-30a mimic, inhibitor or negative control (NC). SNAI1 mRNA levels were significantly downregulated after transfection with the miR-30a mimic and upregulated after transfection with the inhibitor (Fig. 3D). Furthermore, Western blotting revealed that upregulation of miR-30a repressed the protein expression of SNAI1 in LECs, whereas downregulation of miR-30a enhanced the SNAI1 expression (Fig. 3E). Therefore, manipulating the expression of miR-30a-5p affected the expression of SNAI1 in the LECs *in vitro*.

### The inverted regulation of miR-30a on EMT

**Figure 4:**
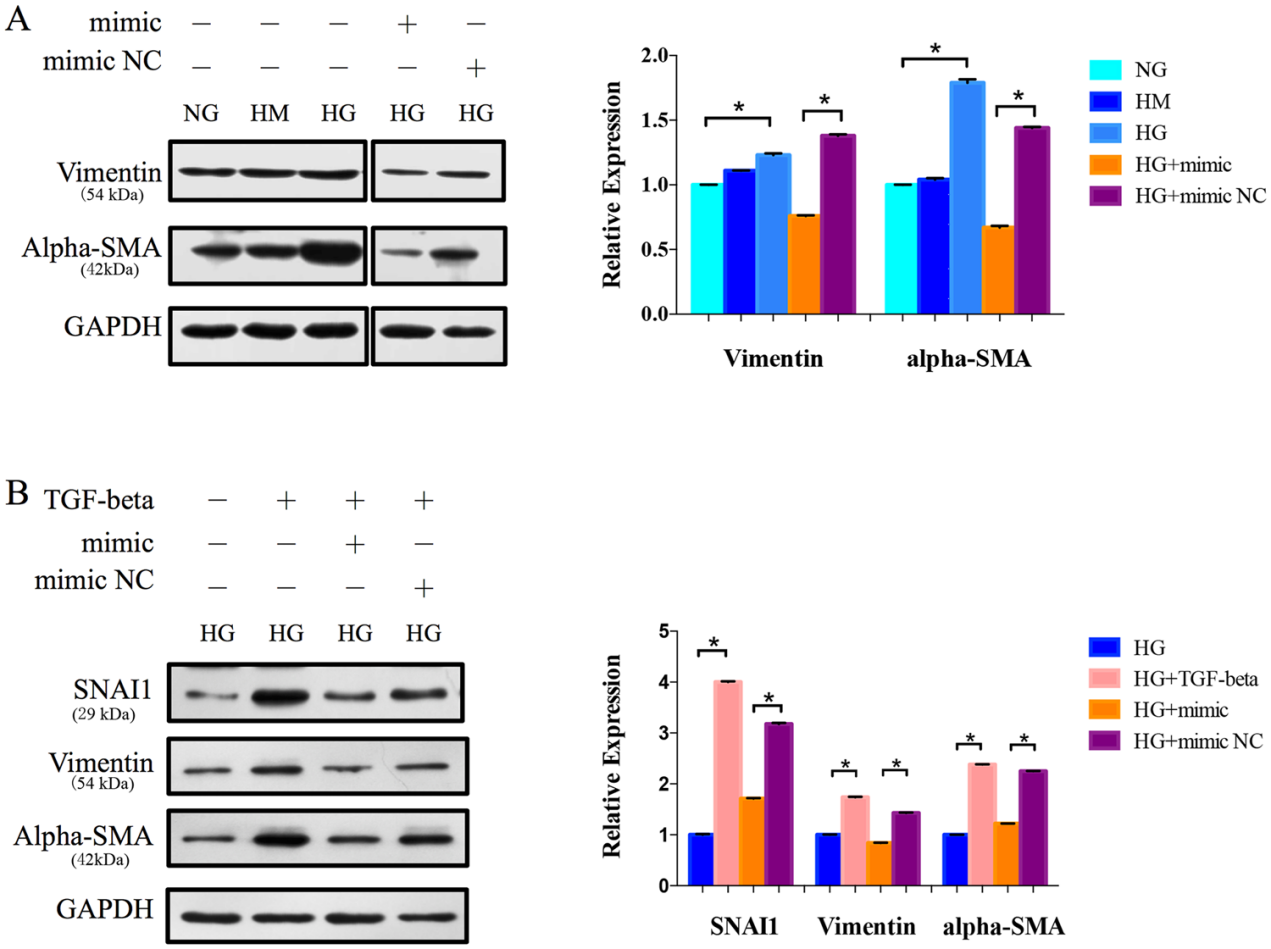
Upregulation of miR-30a-5p repressed EMT. (**A**) Protein expression levels of the EMT markers. The protein expression of vimentin and alpha-SMA was decreased when the LECs were transfected with the miR-30a-5p mimic in HG conditions (*P < 0.05, mimic NC as control, N = 2). (**B**) The capsular bags were treated with TGF-beta and a mimic of miR-30a-5p. In the presence of TGF-beta, EMT was induced with the increased protein levels of vimentin and alpha-SMA, as well as SNAI1 (*P < 0.05, HG without TFG-beta as control, N = 2), while the miR-30a- 5p mimic repressed vimentin and alpha-SMA (P < 0.05, mimic NC as control, N = 2).

To further investigate the role of miR-30a-5p in repressing EMT in the diabetic cataract model *in vitro*, protein levels of vimentin and alpha-SMA were detected. The expression of both vimentin and alpha-SMA proteins was downregulated after the capsular bags were treated with the miR-30a mimic (mimic NC as control, P < 0.05; Fig. 4A). Moreover, the capsular bags were treated with transforming growth factor-beta2 (TGF-beta2) and a miR-30a-5p mimic. In the TGF-beta2 group, alpha-SMA and vimentin were high expressed relative to the group without TGF-beta2 treatment, suggesting an increased EMT level induced by TGF-beta2. By contrast, upregulation of miR-30a decreased the alpha-SMA expression and vimentin expression (Fig. 4B). MiR-30a could downregulate the levels of EMT induced by HG and TGF-beta2 in the diabetic cataract model.

## Discussion

Understanding of the functions of miRNAs on EMT in LECs could provide the means for discovering novel therapies for diabetic cataract. In the present study, miR-30a was observed to be downregulated in diabetic cataract tissues, and miR-30a-5p targeted SNAI1 and inhibited EMT by the SNAI1-dependent way in LECs *in vitro*.

In our previous study^17^, we found that miR-204-5p, miR-30a-5p, miR-204-3p, and miR-184 were significantly downregulated in PCO samples. PCO is a secondary cataract and occurs as an implication of cataract surgery, especially in congenital cataract cases. Although the pathogenesis of different types of cataracts is not well known, the converted expression of miRNAs in PCO gives a sight on diabetic cataract tissues. In the present study, the four miRNAs were also identified to have a low expression in diabetic cataract tissues through the miRNA microarray analysis. The qRT-PCR analysis demonstrated a more significant decrease of miR-30a-5p in diabetic cataract LECs. The expression levels of miRNAs showed partial similarity in capsular bag tissues between PCO and diabetic cataract according to our miRNA microarrays, and miRNAs performed diversified functions by targeting various genes^17–19^. MiRNAs have been reported to be associated with eye development and diseases, such as diabetic corneas^19^, primary angle closure glaucoma^20^, cataract formation^21^, and retinopathy^22^. Many upregulated mRNA transcripts were observed to be candidate targets of the miR-30 family according to the tubulointerstitial fibrosis and gene expression analysis^23^, suggesting that the miR-30 family could be involved in homeostasis. In a previous report, miR-30a was found to target SNAI1, inhibiting invasion and metastasis in non-small cell lung cancer^24^. Therefore, we investigated the role of miR-30a in the regulation of EMT in the diabetic cataract model *in vitro*. To our knowledge, this is the first study to show the importance of miR-30a in the inhibition of lens opacity in diabetic lenses.

EMT has been identified as a significant event in many fibrotic diseases and abnormal differentiation of LECs inducing the formation of cataract^25^. The current researches on EMT mostly focus on the aspect of PCO formation. In a mouse lens capsular injury model, the increased ability of motility and migration of LECs contributed to the formation of lens subcapsular plaque in the injured area^13^. MiR-181a was identified to affect the proliferation and migration abilities of LECs in PCO tissues and inhibit EMT in LECs by targeting c-Met, Slug, and COX-2^26^. In addition, advanced glycation endproducts could enhance the TGF-beta2-mediated EMT during PCO and might participate in aging and diabetes-related fibrosis^11^. Although EMT was an obvious event in age-related cataract tissues^27^, the pathological role of EMT remains unknown in the formation process of diabetic cataract. As vimentin and alpha-SMA are commonly chosen as EMT markers^28–31^, over-productions of vimentin and alpha-SMA in diabetic cataract LECs were found in our study, suggesting that LECs were undergoing EMT in diabetic cataract development. Moreover, induction of SNAI1 expression was noted in majority of instances of EMT^23, 32–34^, and increased SNAI1 levels were correlated with cataract^35, 36^, indicating the important role of SNAI1 in EMT and cataract. In our study, we identified the reverted regulation of miR-30a on SNAI expression by transfecting the miR-30a mimic and inhibitor in LECs. TGF-beta2 has been found to be related to EMT and fibrosis in a number of tissues including ocular tissue, as well as PCO^37, 38^. In the present study, the downregulated expression of EMT markers by miR-30a in LECs was also shown in the presence of TGF-beta2 and HG conditions. We will continue our study on the potential therapeutic role of miR-30a to prevent or/and delay the progress of diabetic cataract.

In conclusion, we identified that EMT is involved in human diabetic cataract, and upregulation of miR-30a can repress EMT through its targeting of SNAI1 in lens epithelial cells, which make miR-30a a novel target of therapeutic intervention for human diabetic cataract.

## Methods

### Lens tissue sample collection

This study was approved by the institutional review board at the Shandong Eye Institute (Qingdao, China). All procedures were performed following the tenets of Declaration of Helsinki. Written informed consent was obtained from each diabetic cataract patient for the collection of samples in the process of cataract surgery by YH. Donor capsular bags were provided by the Qingdao Red Cross Eye Bank (Qingdao, China).

### Human diabetic cataract model *in vitro*

The lens capsular bags were cultured as previously described^17^. Briefly, the capsular bags were put in DMEM-F12 with 10% fetal bovine serum containing 5 mM D-glucose (NG), 25 mM D-glucose (HG), or 5 mmol/L D-glucose plus 20 mmol/L mannitol (HM). After 3 days, they were transfected with a mimic, inhibitor or NC of miR-30a (Guangzhou RiboBio Co., Guangzhou, China) by using our previously reported method^17^. In brief, the miR-30a mimic, inhibitor or NC was mixed with lipofectamineTM 2000 (Invitrogen, Carlsbad, CA) to form miRNA-LipofectamineTM 2000 complexes. Subsequently, the complexes were added in the medium at a final concentration of 50 nM and incubated with the capsular bags for 6 hours. They were also treated with both TGF-beta2) (10 ng/ml) and a miR-30a-5p mimic.

### MiRNA microarray analysis and quantitative RT-PCR

The miRCURYTM LNA array (v.18.0) (Exiqon, Vedbaek, Denmark) was used in this miRNA microarray study (KangChen Bio-tech, Shanghai, China). Three normal samples and three diabetic samples were allocated for this analysis. Total RNA was isolated using a miRNeasy mini kit (QIAGEN), and RNA quality and quantity was measured by using a nanodrop spectrophotometer (ND-1000, Nanodrop Technologies). The miRNA labeling was prepared using the miRCURY™ Hy3™/Hy5™ Power labeling kit (Exiqon), and miRCURYTM LNA array was performed according to the array manual. Scanned images were then imported into GenePix Pro 6.0 software (Axon) for grid alignment and data extraction. For quantitative RT-PCR, total RNA was isolated with a RNeasy micro kit (QIAGEN) from one capsular bag for each group. A MMLV first-strand synthesis kit (Lifetech) and a miRNA first-strand synthesis kit (Clontech, Dalian, China) were used to synthesize the ordinary and micro cDNA. The SYBR Green (Clontech) was used on an ABI 7500 system (Applied Biosystems). The primer sequences used were as follows: SNAI1 F′TTCAACTGCAAATACTGCAACA AG, R′CAGTGTGGGTCC GGACATG. hsa-miR-30a F′TGTAAACATCCTCGA CTGGAA.

### Western blot analysis

Two capsular bags were homogenized in each group, and then the homogenates were assayed with SDS-polyacrylamide gels and transferred to polyvinyl difluoride membranes (Thermo Fisher Scientific). The membranes were stripped and probed with the following primary antibodies: anti-GAPDH (Kangchen), anti-SNAI1 (Abcam), anti-E-Cadherin (BD Transduction Laboratories), anti-Vimentin (Abcam), and anti-alpha-SMA (Abcam). Quantification of Western blots was processed with ImageJ.

### Immunohistochemistry

After incubation with 5% BSA (Boster Biologic Technology) for 30 min at room temperature, paraffin sections of samples were incubated overnight with primary antibodies mentioned above, washed for 15 minutes, and stained with the IHC kit (MXB) at room temperature for 60 min. After washing, a color reaction was detected using a diaminobenzidine kit (ZS-BIO).

### Luciferase activity assay

Human SNAI1-3′UTR containing the putative target site for miR-30a-5p was amplified by PCR and inserted into the pmiR-RB-REPORT (RiboBio), as well as the mutation. The cells were transiently transfected with the wild type or mutant reporter plasmids, miRNA mimic, and NC mimic using Lipofectamine 2000 (Invitrogen). Luciferase activity was measured 48 hours after transfection using the dual-luciferase assay system (Promega). Three independent experiments were performed in triplicate.

### Statistical analysis

Each experiment was performed three times. For descriptive statistics, data are expressed as mean ± standard deviation. Analysis of differential expression was performed using an unpaired t-test. A P value of <0.05 was considered to be statistically significant.
